# A sharp decrease of Th17, CXCR3^+^-Th17, and Th17.1 in peripheral blood is associated with an early anti-IL-17-mediated clinical remission in psoriasis

**DOI:** 10.1093/cei/uxac069

**Published:** 2022-08-04

**Authors:** Sotirios G Tsiogkas, Athanasios Mavropoulos, Efthimios Dardiotis, Efterpi Zafiriou, Dimitrios P Bogdanos

**Affiliations:** Department of Rheumatology and Clinical Immunology, Faculty of Medicine, University of Thessaly, Larissa, Greece; Department of Rheumatology and Clinical Immunology, Faculty of Medicine, University of Thessaly, Larissa, Greece; Department of Neurology, Faculty of Medicine, University of Thessaly, Larissa, Greece; Department of Dermatology, Faculty of Medicine, University of Thessaly, Larissa, Greece; Department of Rheumatology and Clinical Immunology, Faculty of Medicine, University of Thessaly, Larissa, Greece

**Keywords:** anti-IL-17, IL-17, inflammation, psoriasis, T cells

## Abstract

Psoriasis—an immune-mediated skin disease—implicates in its pathophysiology by circulating pro-inflammatory cell populations, cytokines, and their interactions with the epidermis. The direct effect of approved anti-interleukin- (IL-)17A and anti-IL-17R biologic therapy on immunophenotyping of peripheral blood mononuclear lymphocytes’ (PBMCs) relative sub-population frequencies in psoriasis patients has not yet been described. Using multiparameter flow cytometry we examined T-cell subpopulations characterized by CCR6, CCR4, and CXCR3 chemokine receptor surface expression at baseline and after initiation of biologic therapy in PBMCs collected from 30 psoriasis patients. Increased CD3^+^CD4^+^CXCR3^+^, CD3^+^CD4^+^CCR6^+^CCR4^+^CXCR3^+^(CXCR3^+^-Th17), and CD3^+^CD4^+^CCR6^+^CCR4^-^CXCR3^+^(Th17.1) cell populations were observed in patients with psoriasis in comparison to healthy individuals (*n* = 10). IL-17 therapeutic blockade decreased CD3^+^CD4^+^CCR6^+^, CD3^+^CD4^+^CXCR3^+^, CD3^+^CD4^+^CCR6^-^CXCR3^+^(Th1), CD3^+^CD4^+^CCR6^+^CCR4^+^(Th17), CD3^+^CD4^+^CCR6^+^CCR4^+^CXCR3^+^(CXCR3^+^-Th17), and CD3^+^CD4^+^CCR6^+^CCR4^-^CXCR3^+^(Th17.1) cell populations in responding psoriasis patients. Moreover, CD3^+^CD4^-^CCR6^+^, CD3^+^CD4^-^CXCR3^+^, CD3^+^CD4^-^CCR6^+^CCR4^+^(Tc17), and CD3^+^CD4^-^CCR6^-^CXCR3^+^(Tc1) percentages were also inhibited. Modulation of the same cell sub-populations was also assessed in patients treated with methotrexate (*n* = 4), apremilast (*n* = 4), and anti-IL-23 biologic treatment (*n* = 4). In our study, the levels and functional capacity of peripheral pro-inflammatory Th1, Th17, and additional CCR6^+^T cell sub-gated populations from psoriasis patients that were treated with anti-IL-17 or anti-IL-17R targeted biologic therapy were explored for the first time. Our data clearly demonstrate that early anti-IL-17 mediated clinical remission is accompanied by a significant decrease of Th1, Th17, CXCR3^+^-Th17, and Th17.1 cells.

## Teaser

Psoriasis is induced by circulating pro-inflammatory cell populations and the IL-23/Th17 pathway has been arguably recognized as a crucial component for the development of the disease. In psoriasis patients receiving biologic IL-17 therapeutic blockade circulating Th1, Th17 and sub-populations as CXCR3+-Th17 and Th17.1 were decreased as early as 3 months after treatment initiation. Early anti-IL17 mediated clinical remission is accompanied by a significant decrease of specific circulating pro-inflammatory cell populations in therapy-responding patients.

## Introduction

Psoriasis is an immune-mediated skin disease. Psoriasis vulgaris is identified by plaque-type clinical manifestations of the skin [1]. The pathogenesis of the disorder implicates various circulating pro-inflammatory cell populations, cytokines, and their interactions with epidermis, which in term promote keratinocyte hyperproliferation and dysfunctional differentiation [2, 3]. Although, the exact mechanism underlying chronic inflammation is not completely understood, immune dysregulation involving the predominance of effector cell populations has been considered of paramount importance for the pathological processes that eventually contribute to the pathogenesis of the disease [4].

In the early stages, immuno-mediated destruction is promoted by IFNa producing plasmacytoid dendritic cells, which favor skewing of myeloid dendritic cells (mDCs) towards a pro-inflammatory DC phenotype, which expresses both tumor necrosis factor (TNF) and interleukin (IL)-23. Such skewing induces T-cell differentiation toward T helper (Th) 17 and T cytotoxic (Tc) 17 subsets. Moreover, T-cell production of IFNγ has been demonstrated to be involved in the pathogenic cascade of the disease. Furthermore, the IL-23/IL-17 inflammation pathway promotes keratinocyte activation and recruitment of T effector subsets. For example, keratinocyte-produced C–C motif chemokine ligand (CCL−) 20 induces migration of C–C motif chemokine receptor (CCR−) 6 expressing, IL-17 producing T cells in the epidermis, while expression of C–X–C motif chemokine ligand (CXCL-) 9 and CXCL10 among other chemokines by keratinocytes, promotes C–X–C motif chemokine receptor (CXCR-) 3 producing T-cell accumulation. The chemokine-attracted T populations in the skin further produce IL-17, establishing a loop that enhances IL-17 signaling. Activation of dendritic cells, pro-inflammatory cytokine production, T cell subset differentiation, and accumulation in epidermis and chemokine production by triggered keratinocytes concomitantly induce the development of psoriatic skin lesion.

While it may be well established that Th17 cells are of paramount importance for the elicitation of psoriasis, a variety of studies demonstrates how not all Th17 cells are participating equally in inflicting damage [5, 6]. The combined production of both CCR6 and CCR4 surface markers characterizes IL-17 producing CD4^+^ T cells selectively (Th17 cells), whereas co-expression of both chemokine receptors CCR6 and CXCR3 identifies a heterogenous population of cells that produce IL-17 and IFNγ [7]. This IFNγ producing CCR6^+^CXCR3^+^CCR4^−^ (Th17.1 or exTh17) population has been reported to be elevated in bronchoalveolar lavage in sarcoidosis patients [8], to contribute to disease activity in patients with multiple sclerosis [9] and finally predict the response of abatacept treatment in rheumatoid arthritis patients [10]. Other CD4^+^CCR6^+^ subpopulations include double positive CCR4^+^CXCR3^+^ cells (CXCR3^+^-Th17), and double-negative CCR4^−^CXCR3^−^ cells (DN) [11]. Differences in cell surface phenotype characterization within the CD3^+^CD4^+^CCR6^+^ compartment, as shaped by discrete expression of CCR4 or/and CXCR3 markers identify various sub-populations with different immunological behaviors. A meticulous analysis of such cell populations, including their behavior following IL-17 targeted biologic treatment approved for the management of psoriatic patients is needed but to our knowledge has not yet been conducted.

While it is established that various alterations both in the number and the function of T-cell populations that differ between different disease severity phenotypes promote the pathogenesis of psoriasis, only a few studies explore the effect of approved biologic disease-modifying anti-rheumatic drugs (bDMARDs) on immunophenotyping of peripheral blood lymphocytes. On that basis, using flow cytometry we aimed to compare psoriasis-characteristic phenotypes of peripheral T helper and cytotoxic cell populations between 30 patients with psoriasis and 10 healthy controls. To witness not only the alterations of such populations but also the relevance of cell-specific reshaping to treatment’s response, we studied psoriasis patients before and after secukinumab (a human IgG1k monoclonal antibody (mAb) that selectively binds to IL-17A) or brodalumab (a human IgG2 anti-IL-17 receptor A mAb) treatment.

## Patients and methods

### Patients and healthy controls

Participants for this study were patients diagnosed with moderate to severe [Psoriasis Area and Severity Index (PASI) > 7] psoriasis vulgaris (*n* = 30) attending the outpatient clinic of the Department of Dermatology, University General Hospital of Larissa. Our study sample exploring anti-IL-17 therapeutic blockade was composed of 17 males and 13 females with a median age of 52.5 years (interquartile range of 46–63 years). Patients reported onset of the disease at a mean age of 40 years (SD = 12 years). Clinical parameters and patient characteristics are summarized in Table 1. All patients received either secukinumab (*n* = 18) or brodalumab (*n* = 12). Ten age- and sex-matched healthy controls (6 males, 4 females), with a mean age of 49 years were also included in the study. Another cohort of 12 psoriasis patients (8 males and 4 females with a mean age of 49 years) treated with methotrexate (*n* = 4), apremilast (*n* = 4), and anti-IL-23 (*n* = 4) treatment was studied to assess their effect in cell subset changes. The study was conducted in accordance with the Declaration of Helsinki and approved by the Ethics Committee of the University General Hospital of Larissa, University of Thessaly (ECA #19/14-11-2019). Written informed consent was obtained from all subjects involved in the study. Psoriasis disease activity or response to biologic therapy was assessed by PASI score and PASI score improvement, respectively, with a ≥75% improvement accounting for response and a <75% improvement for no response.

**Table 1:** Major demographic and clinical parameters of 30 patients with psoriasis

Patient	Gender	Age	Age of disease’s Onset	Previous Treatment^*^	Washout Period (months)	Current Treatment	Response to treatment	
							Baseline PASI	3 months PASI
1	M	51	48	cDMARDS, apremilast	6	Secukinumab	21.6	0
2	M	66	46	Naïve	n/a	Secukinumab	21	0.5
3	M	53	29	Cyclosporine, retinoids	n/a	Secukinumab	20	0
4	M	53	41	Naïve	n/a	Secukinumab	11.9	0
5	M	65	55	Cyclosporine, retinoids	n/a	Brodalumab	18.5	1.4
6	F	38	31	Naïve	n/a	Brodalumab	10	0
7	M	52	48	Etanercept	34	Brodalumab	12.8	1.7
8	M	70	60	Ustekinumab, secukinumab, apremilast	1	Brodalumab	10	20
9	F	37	26	cDMARDS	n/a	Brodalumab	8.1	0.5
10	M	49	43	Retinoids, cDMARDS	n/a	Secukinumab	11.6	1.5
11	M	64	25	Naïve	n/a	Brodalumab	25.5	2.3
12	M	67	60	Apremilast	0	Brodalumab	30	2.8
13	M	67	53	Adalimumab, cDMARDS, etanercept, cyclosporine, apremilast	1	Secukinumab	10.4	0
14	M	44	37	cDMARDS, cyclosporine, etanercept, ustekinumab	1	Secukinumab	14.1	1.2
15	M	45	42	cDMARDS, adalimumab	3	Secukinumab	13.2	0
16	F	63	19	cDMARDS, cyclosporine, etanercept, ustekinumab	0	Secukinumab	11.9	0.5
17	F	57	51	cDMARDS, cyclosporine, retinoids, ustekinumab	0	Secukinumab	21.2	1.8
18	F	27	18	Naïve	n/a	Secukinumab	17	1.2
19	M	61	32	cDMARDS, cyclosporine	n/a	Secukinumab	8.5	0
20	M	41	27	ustekinumab, adalimumab	13	Brodalumab	7.8	0
21	M	50	34	cDMARDS	n/a	Secukinumab	10.9	0.4
22	F	49	36	cDMARDS, etanercept, ustekinumab	0	Brodalumab	8.5	2.8
23	F	52	43	Retinoids, cDMARDs, ustekinumab, secukinumab	1	Brodalumab	13.5	4.6
24	F	39	26	cDMARDS	n/a	Brodalumab	18	4.1
25	F	62	29	cDMARDS	n/a	Secukinumab	8	1.8
26	F	54	48	Naïve	n/a	Secukinumab	26.6	2.2
27	F	48	38	cDMARDS, etanercept	1	Secukinumab	13.5	1.2
28	F	57	35	ustekinumab	5	Brodalumab	7.2	0
29	F	66	46	cDMARDS, adalimumab	0	Secukinumab	20.5	16.2
30	M	46	60	cDMARDS, etanercept, ustekinumab	1	Secukinumab	11	10.5

Treatment in chronological order.

Abbreviation: PASI, Psoriasis Area and Severity Index; F, female; M, male; naive patient naïve to biologic treatment; cDMARDs conventional disease-modifying anti-rheumatic drugs; washout period months since last biologic or apremilast therapy; n/a patient has not received prior biologic or apremilast treatment.s

Peripheral blood samples (up to 30 ml) were collected from registered participants and layered on the surface of a LymphoPrep gradient (Axis-Shield, Oslo, Norway). Peripheral blood mononuclear cells (PBMCs) were isolated following a protocol including centrifugation and were then washed twice with serum free RPMI 1640 (Pan Biotech). Cell counting was accomplished with a Neubauer hemocytometer and cell viability was determined by trypan blue exclusion, consistently exceeding 95%. PBMCs were then resuspended in cell cryoprotective solution containing 10% dimethyl sulfoxide (DMSO) and 90% fetal calf serum (FCS), aliquoted into cryotubes (Corning, Thermo Fisher Scientific), placed at −80°C for one day, and then stored in liquid nitrogen containers until used.

### Phenotypic analysis of PBMCs by multiparameter flow cytometry

Phenotypic assessment of T cells was performed as previously described [12] using the following anti-human mAbs: fluorescein isothiocyanate (FITC)-conjugated anti-CXCR3 (clone G025H7), phycoerythrin (PE)-conjugated anti-CCR6 (clone G034E3), peridinin chlorophyll protein (PerCP)-conjugated anti-CD3 (clone SK7), allophycocyanin (APC)-conjugated anti-CCR4 (clone L291H4), and PE-Cy7 conjugated anti-CD4 (clone RPA-T4) obtained by BioLegend, San Diego, USA. Isolated PBMCs (0.5–1 × 10^6^ cells) after being washed in phosphate-buffered saline (PBS) were resuspended in staining buffer (containing PBS and 2% FCS), subsequently incubated with labeled mAbs for 30 min on ice, and then fixed with 2% paraformaldehyde solution. Flow cytometry analysis was performed using a Guava EasyCyte 8 (Merck-Millipore, Burlington, USA) cytometer with logarithmic amplification and a forward side light scatter-based gate for total lymphocyte population was applied. To ensure accurate measurement of infrequent T-cell subsets, at least 3 × 10^5^ events were collected within the lymphocyte gate for each sample. Based on isotype control mAbs false-positive and high background readouts were excluded. Results reproducibility was assessed by performing experimental testing at least in duplicate. Data acquisition was achieved using BD Bioscience CellQuest software and data were analyzed with FlowJo, version 10.0.7 (BD Biosciences).

### Detection of intracellular IFNγ and IL-17 expression by T cells

To measure intracellular IFNγ and IL-17A production by T-cell subsets, cells were either left untreated or stimulated with PMA (50 ng/ml) plus ionomycin (1 μg/ml) in the presence of Brefeldin A (10 μg/ml) in RPMI medium containing 10% FCS for 6 h, as previously described [13]. Activated PBMCs were surface stained using FITC-conjugated anti-CXCR3 (clone G025H7), PE-conjugated anti-CCR6 (clone G034E3), APC-conjugated anti-CCR4 (clone L291H4) and PE-Cy7-conjugated anti-CD4 (clone RPA-T4) mAbs, fixed and then permeabilized using commercially available Perm Wash Buffer (BioLegend, San Diego, USA). IFNγ and IL-17A were detected using APC-Cy7-conjugated anti-IFNγ (clone 4S.B3) and PerCP-conjugated anti-IL-17A (clone BL168) mAbs, respectively (obtained by BioLegend, San Diego, USA).

### Magnetic cell isolation

Peripheral blood CD4^+^ T lymphocytes were purified by either positive or negative magnetic cell selection using CD4 mAb coated microbeads and a CD4^-^T cell negative isolation kit, respectively, as previously described. Every cell sorting procedure was performed according to manufacturer’s instructions and yielded >90% pure CD4^+^ T lymphocytes, as consistently evaluated by fluorescence-activated cell sorter (FACS) staining.

### Statistical analysis

Descriptive analysis of our data (counts, means, or medians whenever appropriate) was used to describe the characteristics of the study sample. Percentages of cells expressing cell surface epitopes and MFI values were presented as mean ± SD or median and interquartile range (IQR) whenever appropriate for each group. Differences between the control and patient group, and between different time points in the patient group were assessed, once data were checked for normality by Shapiro–Wilk test, performing the indicated test. Differences between groups are reported by mean values and 95% CI or median values of difference, depending on the type of comparison (parametric or non-parametric test). Simple linear regression was used to assess the effect of previous treatment received on cell inhibition. *P* ≤ 0.05 was considered significant. Statistical calculations were performed using GraphPad Prism, version 9 software.

## Results

In total, 25 of the 30 psoriasis patients achieved a ≥75% improvement in PASI score at 3 months after secukinumab or brodalumab initiation. Of the five non-responding patients, four achieved a therapy-mediated decrease (<75%) in PASI score. In one patient PASI score was further increased 3 months after therapy initiation. Significant differences in routine laboratory lymphocyte analysis at baseline and at 3 months were not observed (Supplementary Table S1).

### Increased percentages of CD3^+^CD4^+^CXCR3^+^ T cells, and CXCR3 expression among CD3^+^CD4^+^CCR6^+^CCR4^−^ and CD3^+^CD4^+^CCR6^+^CCR4^+^ lymphocyte subsets in psoriasis patients compared to healthy controls

The gating strategy of T lymphocyte subsets used for T-cell phenotyping is shown in Supplementary Fig. S1. Supplementary Fig. S1A shows representative flow cytometry plots revealing subdividing strategy for lymphocytes, CD4^+^ T cells, CD4^+^CCR6^+^ cells, CD4^+^CCR4^+^ cells, CD4^+^CXCR3^+^ cells, CD3^+^CD4^+^CCR6^+^CCR4^+^(Th17), CD3^+^CD4^+^CCR6^+^CCR4^+^CXCR3^+^(CXCR3^+^-Th17) and CD3^+^CD4^+^CCR6^+^CCR4^+^CXCR3^−^ (CXCR3^−^-Th17) cells, CD3^+^CD4^+^CCR6^+^CCR4^-^CXCR3^+^(Th17.1) and CD3^+^CD4^+^CCR6^+^CCR4^-^CXCR3− (DN) cells. CD3^+^CD4^-^CCR6^+^CCR4^+^(Tc17) cells were sub-gated accordingly. Supplementary Table S2 summarizes the phenotyping characterization of cell subsets and their matching names.

**Figure 1:**
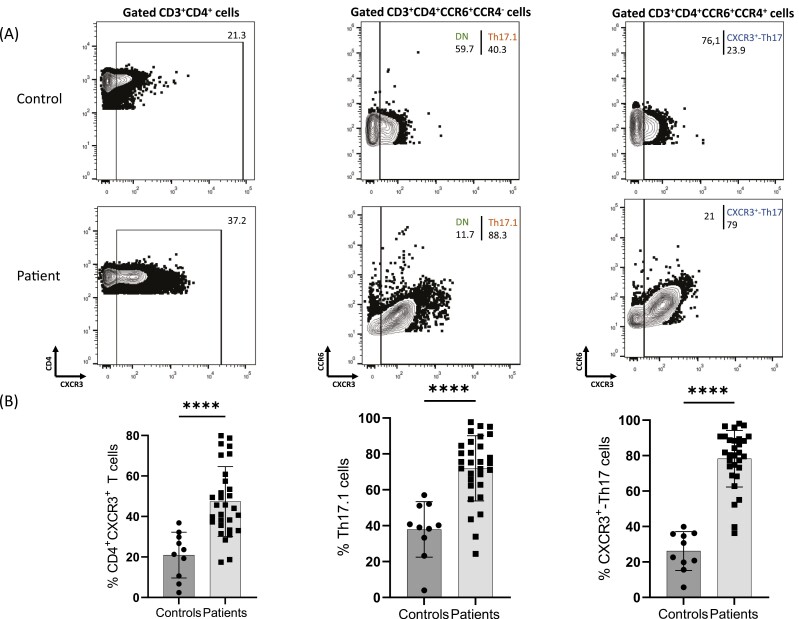
Analysis based on flow cytometry plots from peripheral blood mononuclear cells (PBMCs) from healthy controls (*n *= 10), and psoriasis patients (*n *= 30) was performed. Individual cell subsets were sub-gated according to the expression of CD3, CD4, CCR6, CCR4, and CXCR3 surface markers. **(A)** Representative flow cytometric plots show increased frequencies of Th17.1 and CXCR3^+^-Th17 populations in psoriatic patients compared to healthy controls. **(B)** Box graphical representation showing different frequencies of CD3^+^CD4^+^CXCR3^+^, Th17.1, and CXCR3^+^-Th17 between patients with psoriasis and healthy controls. Percentages out of origin population from which each population is further sub-gated, as described in Supplementary Fig. S1. Bar graphs showing the mean ± SD. ****P ≤ 0.0001 by Mann–Whitney test or unpaired *t*-test.

Figure 1A shows representative flow cytometry plots of CD4^+^CXCR3^+^ T population, Th17.1 and CXCR3^+^-Th17 subsets from a healthy control and a psoriatic patient. Analysis of PBMC flow cytometry at baseline shows an increase (mean = 26.5, 95% CI 14.6–38.3) in the frequency of CD4^+^CXCR3^+^ T cell subset in patients (mean = 47.4, SD = 17.2) in comparison to healthy controls (mean = 20.9, SD = 11.3), *P* < 0.0001. Th17.1 cell percentage is increased (mean = 34.1, 95% CI 21.1–47) in patients (mean = 71.9, SD = 18.2) compared to healthy individuals (mean = 37.9, SD = 15.4), *P* < 0.0001 and CXCR3^+^-Th17 cell percentage is also increased (difference between medians 54.4) in psoriasis patients (mean = 78.3, SD = 16) compared to healthy controls (mean = 26.2, SD = 11), *P* < 0.0001 (Fig. 1B).

### IL-17A therapeutic blockade decreased CD3^+^CD4^+^CCR6^+^ and CD3^+^CD4^+^CXCR3^+^ T-cell sub-populations in psoriasis patients

**Figure 2:**
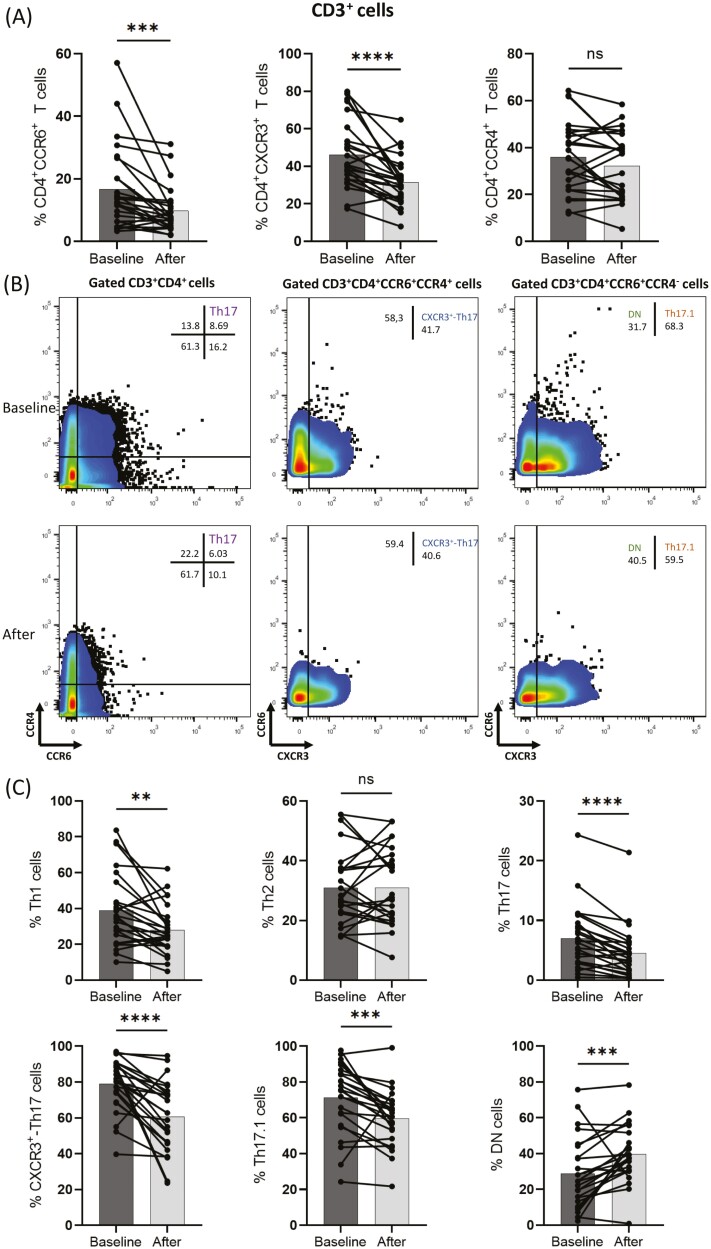
Analysis based on flow cytometry plots from peripheral blood mononuclear cells (PBMCs) from responding to therapy psoriasis patients (*n *= 25) was performed. Individual cell subsets were sub-gated based on the expression of CD3, CD4, CCR6, CCR4, and CXCR3 surface markers. **(A)** Changes in frequency of CD3^+^CD4^+^CCR6^+^T cells, CD3^+^CD4^+^CXCR3^+^T cells, and CD3^+^CD4^+^CCR4^+^T cells in peripheral blood at baseline and after anti-IL-17 biologic therapy. The percentage out of CD4 population is displayed. **(B)** Representative flow cytometric plots showing changes in T-cell phenotypes in a psoriasis patient at baseline and after anti-IL17 biologic therapy. **(C)** Box graphical representation showing a significant decrease in Th1, Th17, CXCR3^+^-Th17, and Th17.1 cell subsets after anti-IL17 biologic therapy and an increase in DN cells in peripheral blood from psoriasis patients. Percentages out of origin population from which each population is further sub-gated, as described in Fig. 1. Bar graphs showing the mean ± SD. ns *P* > 0.05, **P ≤ 0.01, ***P ≤ 0.001, ****P ≤ 0.0001 by Wilcoxon signed rank test or paired *t*-test.

As shown in Fig. 2A, in responding patients secukinumab and brodalumab treatment decreased (median of difference −3.3) the percentage of CD3^+^CD4^+^CCR6^+^ population after treatment (mean = 9.8, SD = 7.3) compared to baseline (mean = 16.7, SD = 13.4), *P* = 0.0001. The percentage of CD3^+^CD4^+^CXCR3^+^ T cells was also significantly decreased (median of differences −10.20) in responding patients at 3 months of intervention (mean = 31.6, SD = 12.6) compared to baseline (mean = 46.2, SD = 18.1), *P* < 0.0001. CD3^+^CD4^+^CCR4^+^cells did not differ significantly between before and after treatment.

### IL-17 therapeutic blockade decreased Th1, Th17 cell populations and expression of CXCR3 among CD4^+^CCR6^+^CCR4^+^ and CD4^+^CCR6^+^CCR4^−^ cell subsets in psoriasis patients

Supplementary Fig. S1B shows representative flow cytometry plots revealing subdividing strategy for CD3^+^CD4^+^CCR6^−^CXCR3^+^(Th1) and CD3^+^CD4^+^CCR6^-^CXCR3^−^CCR4^+^(Th2). CD3^+^CD4^−^CCR6^−^CXCR3^+^(Tc1) and CD3^+^CD4^-^CCR6^-^CXCR3^−^CCR4^+^(Tc2) cells were gated accordingly.

Figure 2B shows representative flow cytometry plots of Th17, CXCR3^+^-Th17, and Th17.1 cells from a psoriasis patient at baseline and after 3 months of biologic treatment. In general, the percentage of Th17, CXCR3^+^-Th17, Th17.1, and Th1 was significantly decreased in responding to therapy psoriasis patients following treatment compared to baseline. More specifically, the initial Th17 cell percentage mean = 7 (SD = 5.2) decreased (mean = −2.4, 95% CI −3.3 to −1.5) after therapy to mean = 4.6 (SD = 4.4), *P* < 0.0001, the initial CXCR3^+^-Th17 cell percentage mean = 78.9 (SD = 14.6) also decreased (median of difference −17.5) to mean = 60.6 (SD = 19.6), *P* < 0.001 and the initial Th17.1 cell percentage mean = 71.2 (SD = 19.4) decreased (median of difference −11.3) to mean = 59.7 (SD = 15.5), *P* = 0.002 after intervention, whereas the percentage of Th2 cells remained largely unchanged (Fig. 2C).

### IL-17 therapeutic blockade decreased CD3^+^CD4^−^CCR6^+^, CD3^+^CD4^−^CXCR3^+^, Tc1, and Tc17 cell populations in psoriasis patients

**Figure 3:**
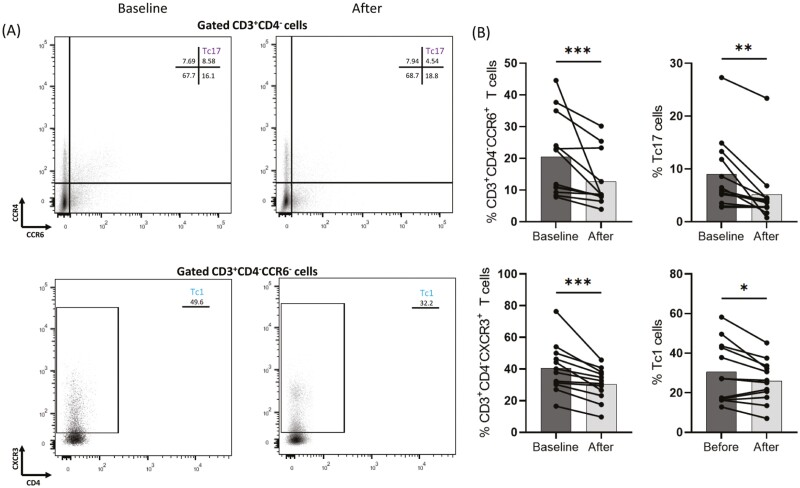
Analysis of CD3^+^CD4^−^ sub-population based on flow cytometry plots from peripheral blood mononuclear cells (PBMCs) from responding psoriasis patients was conducted (*n *= 12). Individual cell subsets were sub-gated based on the expression of CD3, CD4, CCR6, CCR4, and CXCR3 surface markers. **(A)** Representative flow cytometric plots showing changes in T-cell phenotypes in a psoriasis patient at baseline and after anti-IL17 biologic therapy. **(B)** Box graphical representation showing a significant decrease in CD3^+^CD4^−^CCR6^+^T, CD3^+^CD4^-^CCR6^+^CCR4^+^(Tc17), CD3^+^CD4^−^CXCR3^+^T, and CD3^+^CD4^−^CCR6^-^CXCR3^+^(Tc1) cell subsets after anti-IL17 biologic therapy in peripheral blood from psoriasis patients. Percentages out of origin population from which each population is further sub-gated. Bar graphs showing the mean ± SD. *P ≤ 0.05, **P ≤ 0.01, ***P ≤ 0.001 by Wilcoxon signed rank test or paired t-test.

Figure 3A shows representative flow cytometry plots of Tc17 and Tc1 cells from a psoriasis patient at baseline and after 3 months of anti-IL-17 biologic treatment. As shown in Fig. 3B anti-IL17A and anti-IL17R biologics decreased (median of difference −3.9) the percentage of CD3^+^CD4^-^CCR6^+^T cells after therapy (mean = 12.7, SD = 8.6) compared to baseline (mean = 20.5, SD = 12.8), *P* = 0.001. The percentage of CD3^+^CD4^−^CXCR3^+^T cells was also significantly decreased (median of difference −8.1) in patients after biologic intervention (mean = 30.5, SD = 10.1) compared to baseline (mean = 40.5, SD = 15.5), *P* = 0.0005. Moreover, biologic therapy decreased (mean = −4.6, 95% CI −8.8 to −0.4) the percentage of Tc1 subset from mean = 30.5 (SD = 15.4) to mean = 25.9 (SD = 10.7), *P* = 0.0341 and the percentage of Tc17 subset was also inhibited (mean = −3.9, 95% CI −6.2 to −1.5) from mean = 9 (SD = 7) before therapy to mean = 5.1 (SD = 6) at 3 months after treatment initiation, *P* = 0.0038.

### Effect of previous treatments on cell-subset changes compared to anti-IL17A initiation

We also assessed whether any treatment initiated before anti-IL17A or anti-IL-17R biologic treatment was associated with changes in the percentages of cell subset populations. The analysis included baseline and 3-month results of patients (*n* = 25) subdivided into five groups according to previous treatment: naïve (*n* = 6), on csDMARDs (*n* = 8), apremilast (*n* = 3), etanercept, or adalimumab (anti-TNF) (*n* = 4), and patients who were treated with ustekinumab (*n* = 4) before secukinumab or brodalumab initiation. For Th17 and each cell sub-population (CXCR3^+^-Th17, and Th17.1) a separate univariate linear regression was performed to evaluate the effect of prior therapy (independent variable) on cell-subsets at baseline and at 3 months (dependent variable). The naïve group was used as a reference group for each analysis. As shown in Supplementary Table S3, prior treatment with apremilast affected changes of CXCR3^+^-Th17 percentages. No other statistically significant differences were found.

### Methotrexate, apremilast, and anti-IL-23 treatment also modulate pro-inflammatory cell populations

We also evaluated, in a limited number of samples, whether the effect the of anti-IL17 therapeutic blockade on cell is specific for anti-IL-17 biologics or a similar effect is mediated after treatment of psoriasis patients with other agents. PBMC samples from 12 responders, including 5 naïve to previous therapy, 4 previously treated with csDMARDs, and 3 treated with biologic therapy were assessed at (treatment initiation) and at 3 months of their respective therapy. We assessed the effect of methotrexate (*n* = 4), of apremilast (*n* = 4) and of anti-IL-23 biologic treatment (*n* = 4; guselkumab or risankizumab). As shown in Supplementary Fig. S2 methotrexate treatment inhibited CD4^+^CXCR3^+^(mean = -13, 95% CI -25.12 to -0.88), Th1 (mean = −6.85, 95% CI −10.99 to −2.71) and CXCR3^+^-Th17 (mean = −10.25, 95% CI −16.79 to −3.7) cell frequencies. Apremilast treatment inhibited the percentage of CD4^+^CXCR3^+^(mean = −6.525, 95% CI −8.26 to −4.79) and Th1 cells (mean = −8.6, 95% CI −17.14 to −0.061) but did not affect CXCR3^+^-Th17, while anti-IL-23 biologic treatment decreased the percentage of CD4^+^CCR6^+^(mean = −6.81, 95% CI −13.46 to −0.167), Th17 (mean = −4.84, 95% CI –9.24 to −0.447) and CXCR3^+^-Th17 (mean = −28.76, 95% CI −51.49 to −6.037) cells.

## Discussion

In this study, we explored for the first time the levels and functional capacity of peripheral pro-inflammatory phenotypically characterized as Th17 population (CCR6^+^ and CCR4^+^), Th1 subset (CXCR3^+^ CCR6^−^ and CCR4^−^), and sub-populations of Th17 that have switched into IFNγ producing (Th1 like cells) (CCR6^+^ CCR4^−^ CXCR3+) [14–16] in psoriasis patients at baseline and at 3 months after initiation of treatment with anti-IL-17 or anti-IL-17R targeted biologic therapy. Our data clearly demonstrate that early anti-IL17 mediated clinical remission is accompanied by a significant decrease of Th17, CXCR3^+^-Th17, Th17.1, and Th1 cells.

In our study, we found that CD4^+^CCR6^+^T cells had the most significant decrease after anti-IL17 or anti-IL17R biologic therapy. Recent studies suggest that CD4^+^CCR6^+^ population is heterogeneous [17, 18]. Secukinumab or brodalumab therapy did not affect proportions of CD3^+^CD4^+^ and CD3^+^ CD4^-^ populations (Supplementary Fig. S3), whereas treatment significantly decreased Th17, Th17.1 CXCR3^+^-Th17 cells and altered their relative distribution in origin population CD4^+^CCR6^+^ after therapy (Supplementary Fig. S4). Such changes were not found in non-responders (Supplementary Fig. S5). Interestingly, IL-17 targeted therapy affected to a various extent the percentages of these T-cell populations, but intracellular IL-17 production also differed between these cell subsets in psoriasis patients as shown in Supplementary Fig. S6, further indicating that the immunophenotypic characteristics of those cells and their ability to be affected by treatment-induced IL-17 inhibition is diverse and must be characterized further at the functional level.

**Figure 4:**
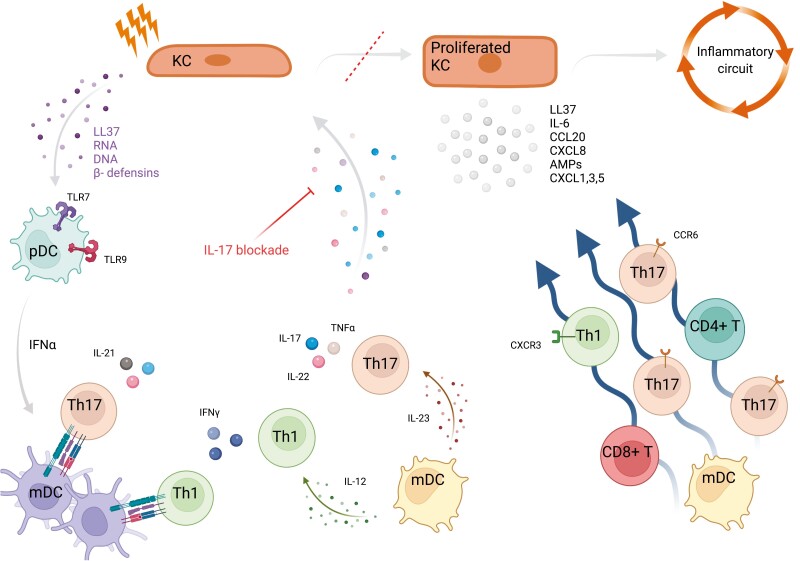
A schematic representation of pro-inflammatory events facilitated during the establishment of interleukin- (IL-) 17 mediated psoriasis feed-forward circuit. Skin damage induces the release of self-nucleotides. Complexed self-nucleotides with antimicrobial peptides (AMPs) are recognized by Toll-like receptors (TLRs) of plasmacytoid dendritic cells (pDCs). Interferon (IFN) α produced, stimulates myeloid dendritic cells (mDCs), which express T helper (Th) 17 and Th1 differentiating cytokines. Furthermore, cell to cell interactions boost differentiating signals. Th17 produces IL-17, IL-21, IL22, tumor necrosis factor (TNF) α, among various cytokines that promote keratinocyte proliferation. Keratinocyte proliferation amplifies IL-23-producing signals, resulting in release of pro-inflammatory cell-attracting chemokines, thus inducing the inflammatory circuit established in psoriasis. Treatment with either anti-IL17A or anti-IL17RA biologics interrupts the IL-17-dependent inflammatory loop. KC, keratinocyte. Created with BioRender.com, under license to DPB.

Our study cannot provide a mechanistic explanation for the observed changes in cell subset percentages other than that which is logical to assume i.e. that directly or indirectly those changes are mediated by IL-17 inhibition. Previous studies have shown that the pathophysiology of psoriasis involves the establishment of a pro-inflammatory loop that starts with keratinocyte damage induced by trauma, infection, or drugs [19]. As a result, complexes of self-nucleic acids with AMPs, such as LL37 (cathelicidin), β-defensins, and S100 or with IL-26 derived from Th17 cells have been reported to activate pDCs, through TLR7, TLR8, and/or TLR9 stimulation [20]. Activated pDCs produce type I interferons, which promote mDC maturation and Th differentiation [21, 22]. The two populations of psoriasis-associated mDCS, which are suggested to originate from precursors in circulation, upon activation produce IL-23, TNFα, IL-6, and IL-12 driving differentiation of T cells toward a Th17 and/or Th1 phenotype, and also present antigens to T cells establishing structures in the dermis that resemble lymphoid tissue [23, 24]. Interestingly, the formation of such dermal clumps is heavily relied on the CCL20/CCR6 chemokine system [25]. Skewing of T cells towards a Th17, a Th1, or a Th1-like Th17 phenotype results in IL-17, IL-21, IL-22, and IFNγ production [24, 26]. Keratinocytes respond to such signals by chemokine (including CCL20 and CXCL8-11), AMP, and cytokine (including IL-6, TNF-a) production [27, 28]. Subsequently, further CCR6^+^ dendritic and T-cell subset recruitment and Th17 differentiation are established, which amplify IL-17-mediated proinflammatory signals [29]. Such a feed-forward inflammatory loop has been proposed to be facilitated in psoriasis [20, 30]. Though we cannot speculate with confidence, it could be possible the therapeutic blockage of IL-17A or IL-17R may exert an inhibitory effect on keratinocyte proliferation (Fig. 4). Blocking IL-17, a cytokine that appears to be crucial for the maintenance of the pro-psoriatic circuit, decreases among others CCL20 and CXCL9-10 levels. That inhibits inflammatory dendritic and T cell dermal accumulation, hinders pro-Th17 differentiation signals, and attenuates loop-amplifying AMP and cytokine (IL-23, IL-12, and IL-6) production.

While consensus regarding Th17 differentiation has not yet been achieved, IL-23, IL-1, and IL-6 have been considered instrumental for such a process to take place. TNFα has also been reported to possess indirect Th17 activating capacities [31]. Those cytokines have been reported to upregulate RORγt, thus inducing a Th17 phenotype and IL-17 production [16, 32–34]. Although, the model of STAT3/RORγt mirrors the traits of Th17 differentiation, it fails to reflect its complexity, since Th17 has been recognized as a population with high plasticity [35]. Specifically, exposure to IL-12 induces Th17 cells to IFNγ production [36], as it inhibits RORγt and upregulates T-bet [37]. Moreover, differentiation of Th17 into Th1-like populations has been reported to be IL-23-induced [38]. Inhibition of IL-17 mediated amplification of psoriasis inflammatory loop may translate in lower levels of IL-21 and IL-23, affecting the skewing of Th17 populations towards Th1-like cell subsets.

Recently developed biologic therapies that target not only the IL-23-Th17-IL-17 axis but the TNF-α-signaling as well, exhibit high efficacy and safety in patients with psoriasis. Biologically targeted therapies exert their immunosuppressive action by modulating immune responses. Such therapies may revert the established pro-inflammatory milieu through their direct effect on cytokine-producing cell populations. In recent studies, methotrexate has been found to decrease Th17, Th1 cell percentages in blood and restore Treg populations and their immunosuppressive function in patients with psoriasis [39, 40]. Also, small molecule and biologic therapies have been shown to increase Th2, Treg cell populations and decrease Th1, Th9, and Th22 cells in blood of patients after treatment [12, 41]. Previously, our group has demonstrated that apremilast, a phosphodiesterase 4 inhibitor successfully used for the treatment of the disorder, increases IL-10 producing regulatory B cells and decreases IFNγ and IL-17 producing pro inflammatory T subsets in psoriasis and psoriatic arthritis patients [12]. Etanercept has been found to induce apoptosis in dermal dendritic cells [42], but another study failed to illustrate a possible correlation between clinical response and T-cell compartmentalization in peripheral blood or epidermis [43]. Moreover, infliximab also decreased frequencies of circulating Th1 and Th17 cells in a small number of patients [44] in PBMCs but failed to inhibit IL-22 production by epidermal CD4^+^ T cells retained in restored skin six years after treatment initiation [45]. A single study has demonstrated that adalimumab decreases Th1, Th17, and Th22 populations and their associated transcription factors [46], contrasting another study that showed that adalimumab indeed increased Th17 cells in circulation in psoriatic arthritis patients [47]. Of relevance to the present study, recent data indicated that in patients with psoriasis IL-17 and IL-22 producing cells are in a substantial proportion CD3^-^ innate lymphoid cells (ILC), and adalimumab treatment decreased the percentages of circulating NKp44^+^ILC3 [48]. Another study demonstrated that guselkumab and secukinumab decrease inflammatory monocyte-like and dendritic-like cells in lesioned skin [49]. A report examining a PsA patient’s invariant T, Th17, and Th17.1 cells in blood showed no significant difference between populations at baseline compared to after ixekizumab treatment [50].

In a recent report, researchers allocated psoriatic arthritis patients in a standard bDMARD treatment group and a strategic bDMARD treatment group, in which specific a biologic agent was chosen based on phenotypical characteristics of T helper cells as shown by flow cytometry analysis of patients’ blood. Patients allocated in the strategic bDMARD group were accordingly categorized in four types of phenotypical profiles with a matching biologic therapy: dominant Th1 phenotype/ustekinumab, dominant Th17 phenotype/ secukinumab, high Th1, and Th17 phenotype/secukinumab or anti-TNF and low Th1 and Th17 phenotype/adalimumab or infliximab. Patients receiving strategically chosen biologic treatment achieved at 6 months of treatment higher rates of low disease activity (SDAI, DAS28, ACR20) compared to the standard bDMARD treatment group [51]. We herein provide evidence that secukinumab and/or brodalumab most likely reform circulating Th17 cell phenotype profile of psoriasis patients and suggest that clinical remission is associated with cell subset re-shaping. Psoriasis patients with a dominant Th17 and Th1-like Th17 subset profile may be benefited from anti-IL-17 and/or anti-IL-17RA treatment. Moreover, early assessment during treatment by flow cytometry indicative of Th17 cell and sub-populations inhibition may operate as a surrogate marker of subsequent clinical remission.

To assess whether the effect of anti-IL-17 targeted therapy is drug-specific, we explored in a limited number of patients with available PBMCs, the influence of other treatment regimens on cell-subset changes. Methotrexate-treated patients in remission showed a decrease in CD4^+^CXCR3^+^, Th1, and CXCR3^+^-Th17 cell frequencies. Apremilast treatment inhibited the percentage of CD4^+^CXCR3^+^and Th1 cells but did not affect CXCR3^+^-Th17, while anti-IL-23 biologic treatment decreased the percentage of CD4^+^CCR6^+^, Th17, and CXCR3^+^-Th17 cells. Those data imply an effect on various cell subsets, non-specific to anti-IL-17 targeted therapy but more data are needed to shed light on the extent of their influence.

Also, when an analysis was made to assess the effect of previous treatments on cell-subset changes in patients who consequently received anti-IL17A initiation it was found that only apremilast exerted significant changes and this was noted only for CXCR3^+^-Th17 percentages. However, the observed data are not sufficient enough as the number of patients analyzed was relatively small and the interpretation of data must be treated with caution.

Our study demonstrates for the first that T-cell population characterization merely by surface chemokine receptor phenotyping is sufficient to provide a vital information concerning the likely outcome of patients that were treated with IL-17 targeted treatment, irrespectively whether that treatment relates to the inhibition of IL-17A or IL17-RA. Our data should be treated with caution since larger independent studies must be conducted to elucidate whether these cell subsets could be considered as potential patient classifying markers that could be implemented towards more personalized treatment options in the future.

## Supplementary Material

uxac069_suppl_Supplementary_Figure_S1

uxac069_suppl_Supplementary_Figure_S2

uxac069_suppl_Supplementary_Figure_S3

uxac069_suppl_Supplementary_Figure_S4

uxac069_suppl_Supplementary_Figure_S5

uxac069_suppl_Supplementary_Figure_S6

uxac069_suppl_Supplementary_Legends

uxac069_suppl_Supplementary_Table_S1

uxac069_suppl_Supplementary_Table_S2

uxac069_suppl_Supplementary_Table_S3

## Data Availability

The data underlying this article will be shared on reasonable request to the corresponding author.

## Abbreviations

bDMARDs: biologic disease-modifying anti-rheumatic drugs
CCL: C-C motif chemokine ligand
CCR: C-C motif chemokine receptor
csDMARDs: conventional synthetic disease-modifying anti-rheumatic drugs
CXCL: C-X-C motif chemokine ligand
CXCR: C-X-C motif chemokine receptor
IFN: interferon
IL: interleukin
mAb: monoclonal antibody
mDCs: myeloid dendritic cells
PASI: Psoriasis Area and Severity Index
PBMCs: peripheral blood mononuclear lymphocytes
Tc: T cytotoxic
Th: T helper.
