# Supplementary figures and images for: A sharp decrease of Th17, CXCR3+-Th17, and Th17.1 in peripheral blood is associated with an early anti-IL-17-mediated clinical remission in psoriasis
Source: Clin Exp Immunol. 2022 Aug 4;210(1):79–89. doi: 10.1093/cei/uxac069 (PMC9585551; doi:10.1093/cei/uxac069)

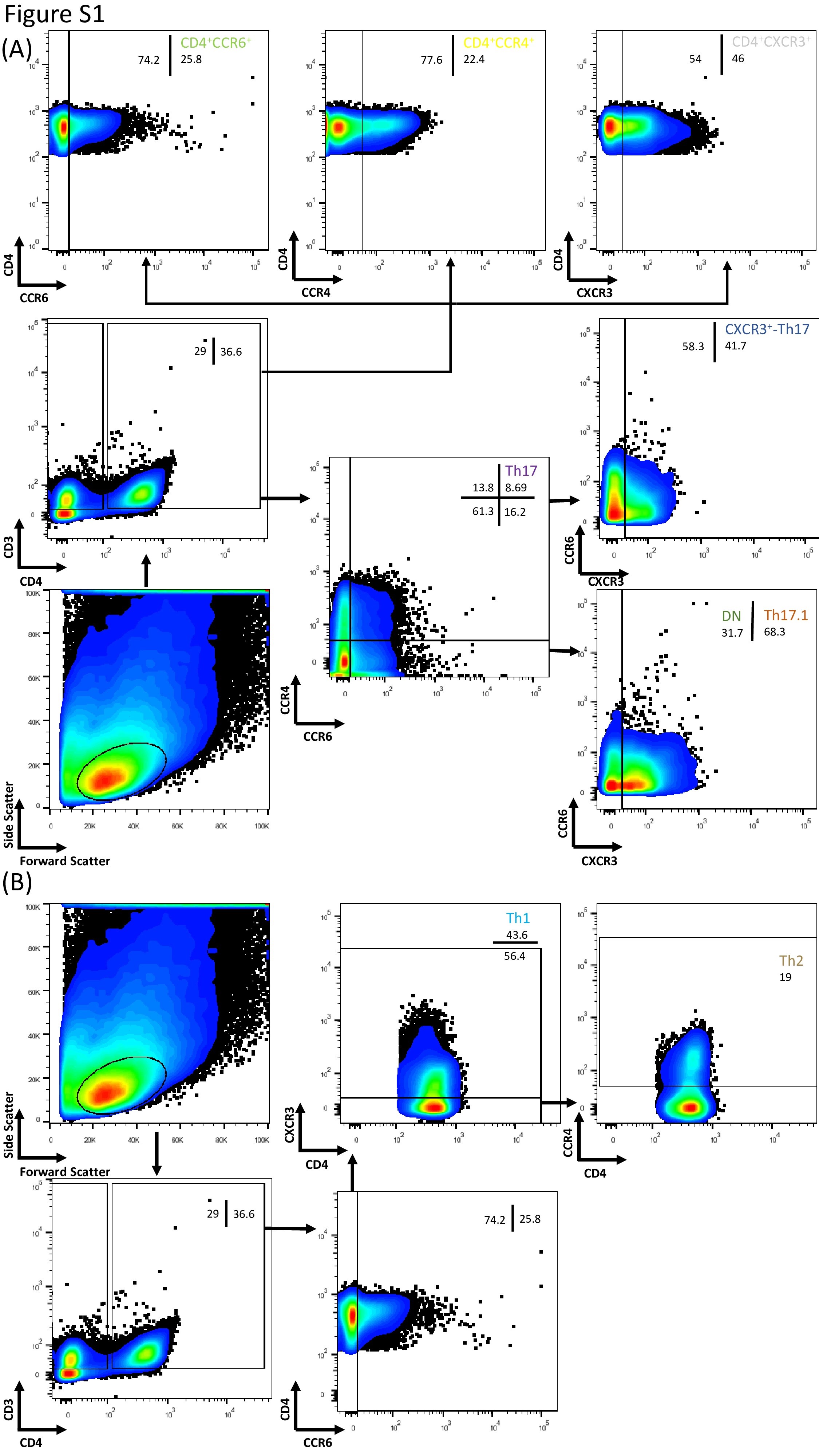

Supplement: uxac069_suppl_Supplementary_Figure_S1 [file uxac069_suppl_supplementary_figure_s1.jpeg]

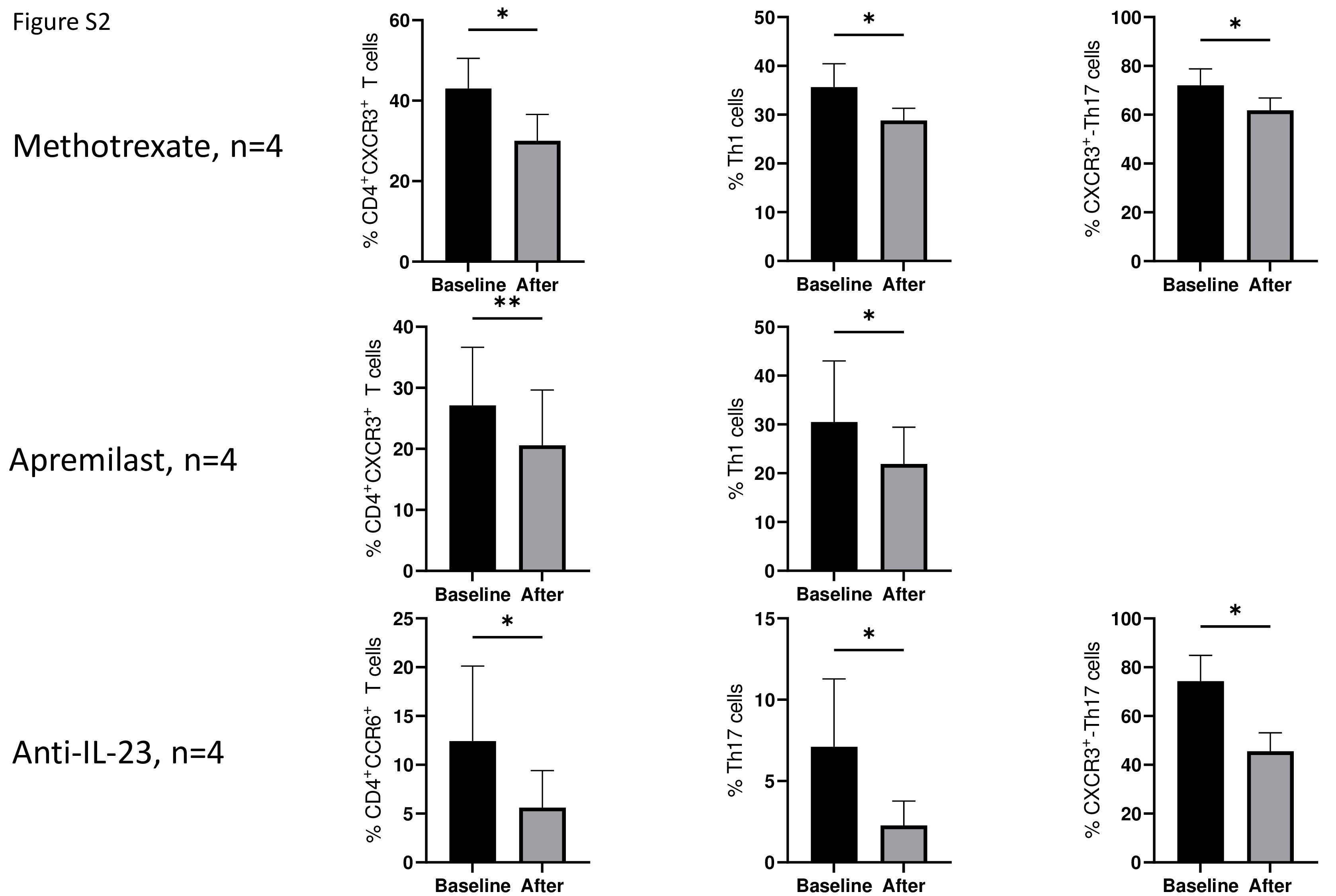

Supplement: uxac069_suppl_Supplementary_Figure_S2 [file uxac069_suppl_supplementary_figure_s2.jpeg]

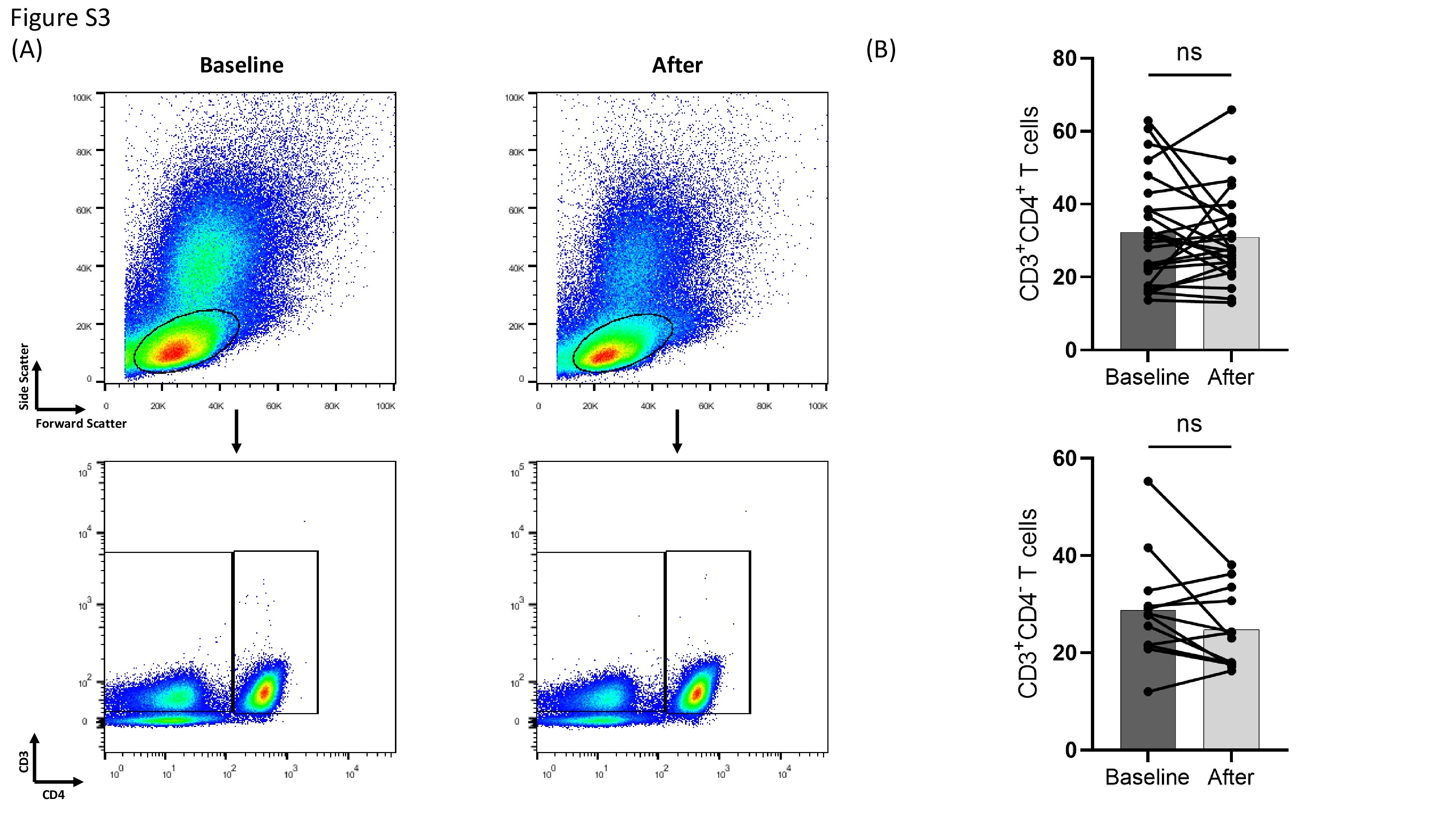

Supplement: uxac069_suppl_Supplementary_Figure_S3 [file uxac069_suppl_supplementary_figure_s3.jpeg]

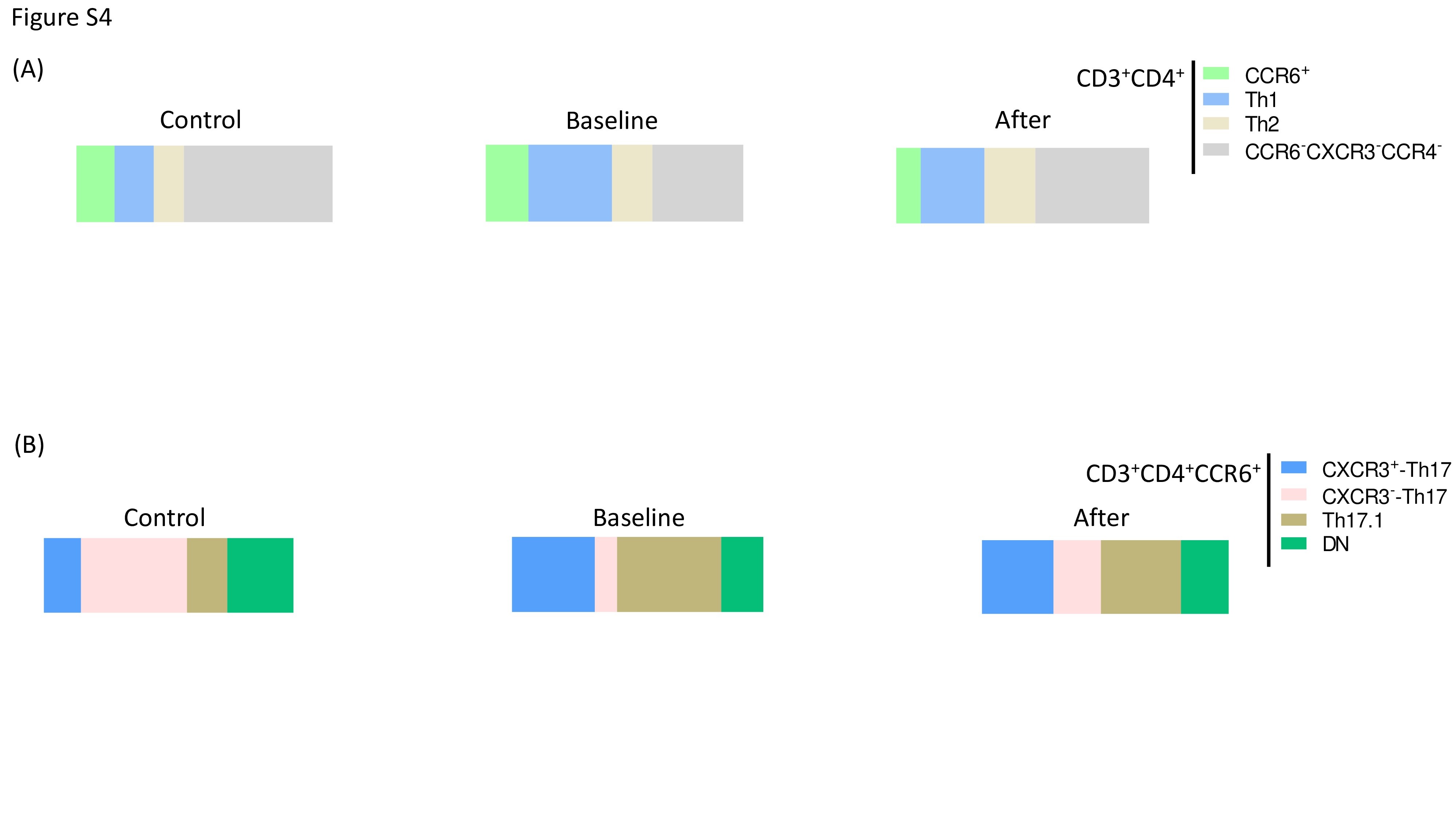

Supplement: uxac069_suppl_Supplementary_Figure_S4 [file uxac069_suppl_supplementary_figure_s4.jpeg]

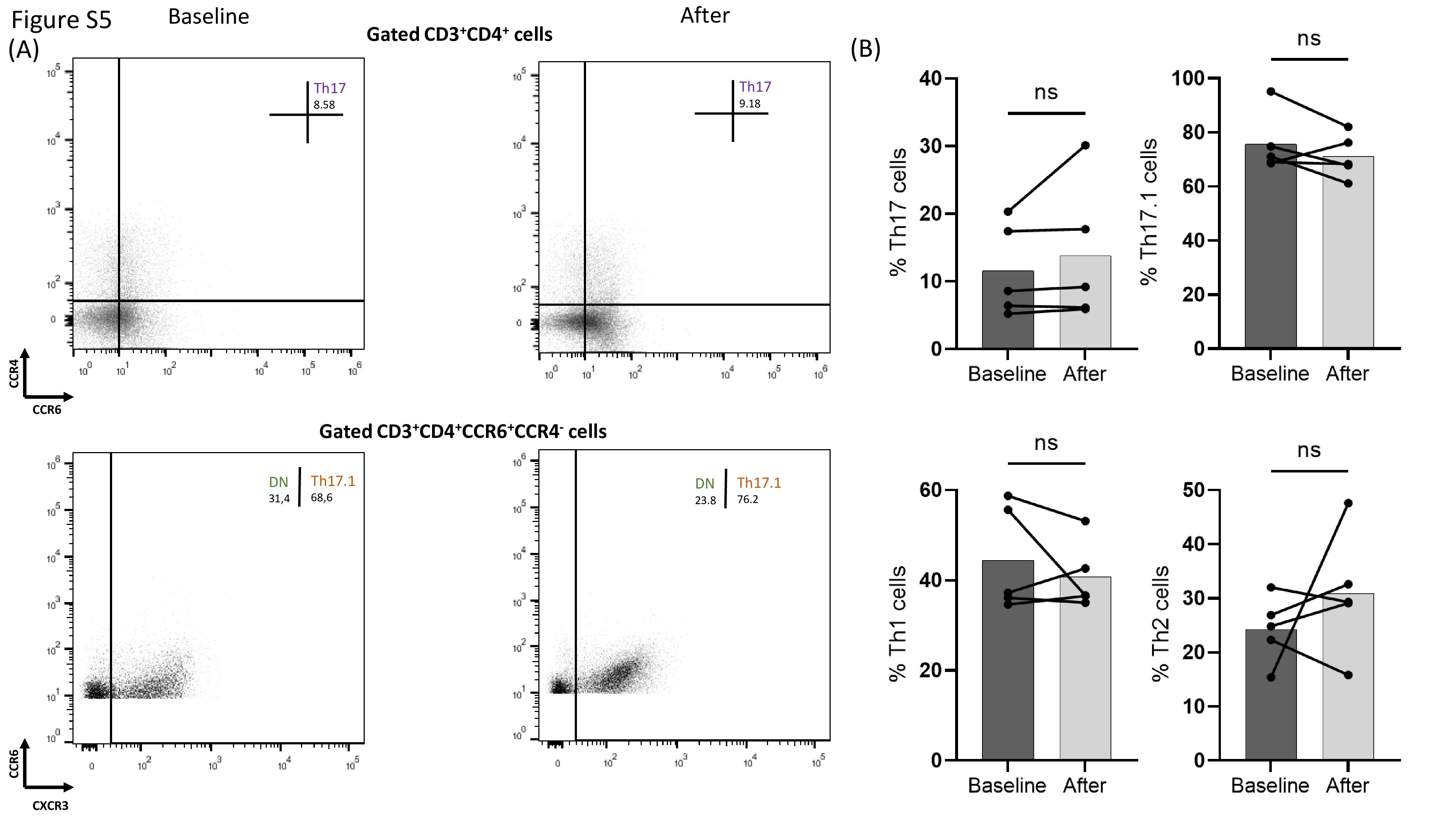

Supplement: uxac069_suppl_Supplementary_Figure_S5 [file uxac069_suppl_supplementary_figure_s5.jpeg]

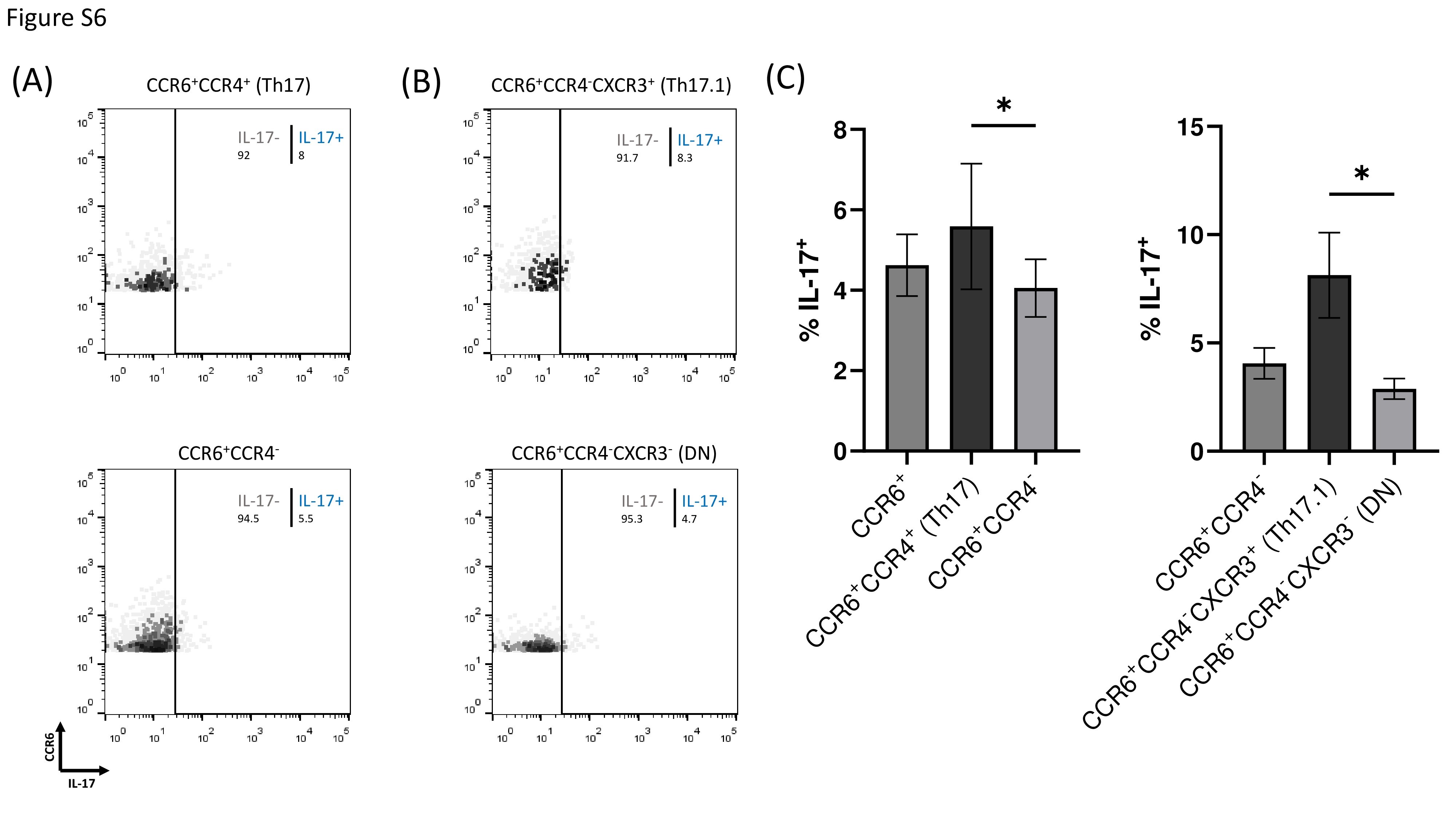

Supplement: uxac069_suppl_Supplementary_Figure_S6 [file uxac069_suppl_supplementary_figure_s6.jpeg]
