# Supplementary material for: A sharp decrease of Th17, CXCR3+-Th17, and Th17.1 in peripheral blood is associated with an early anti-IL-17-mediated clinical remission in psoriasis
Source: Clin Exp Immunol. 2022 Aug 4;210(1):79–89. doi: 10.1093/cei/uxac069 (PMC9585551; doi:10.1093/cei/uxac069)
Supplement: uxac069_suppl_Supplementary_Legends [file uxac069_suppl_supplementary_legends.docx]

**Supplementary Figures Legends**

**Figure S1.** Representative gating strategy of peripheral blood mononuclear cells (PBMCs). **(A)** Flow cytometry plot depicting lymphocytes and derived plots used to gate the populations of interest. CD3^+^CD4^+^ lymphocytes were gated, and within that population expression of CCR6, CCR4 and CXCR3 chemokine receptors was examined. Cells co-expressing CCR6, CCR4 were characterized as T helper (Th) 17, and further characterization by CXCR3 expression to that population was performed. CCR6^+^CCR4^-^ cell population was also examined for CXCR3 expression to identify Th17.1 and double-negative (DN) cells. **(B)** Flow cytometry plot depicting lymphocytes and derived plots used to gate the populations of interest. CD3^+^CD4^+^ lymphocytes were gated, and within that population CCR6^-^ cells were further gated. CCR6^-^ cells expressing CXCR3 were characterized as Th1, while CCR6-CXCR3-CCR4+ cells were determined to be Th2.

**Figure S2.** **(A)** Box graphical representation showing different frequencies of CD3^+^CD4^+^CXCR3^+^, Th1 and CD3^+^CD4^+^CCR6^+^CCR4^+^CXCR3^+^ (CXCR3^+^-Th17) after 3 months of methotrexate treatment in patients with psoriasis (n=4) compared to baseline. **(B)** Box graphical representation showing different frequencies of CD3^+^CD4^+^CXCR3^+^, and Th1 cells after 3 months of apremilast therapy in patients with psoriasis (n=4) compared to baseline. **(C)** Box graphical representation showing different frequencies of CD3^+^CD4^+^CCR6^+^, CD3^+^CD4^+^CCR6^+^CCR4^+^ (Th17) and CD3^+^CD4^+^CCR6^+^CCR4^+^CXCR3^+^ (CXCR3^+^-Th17) after 3 months of risankizumab or guselkumab therapy in psoriasis patients (n=4) compared to baseline. Percentages out of origin population from which each population is further sub-gated, as described in Figure S1. Bar graphs showing the mean ± SD. * p ≤ 0.05, ** p ≤ 0.01 by Wilcoxon signed rank test or paired t-test.

**Figure S3.** Secukinumab or brodalumab therapy did not affect proportions of CD3^+^CD4^+^ and CD3^+^CD4^-^ populations. **(A)** Representative flow cytometric plots showing gated lymphocytes, and CD3^+^CD4^+^, CD3^+^CD4^-^ subpopulations of a psoriasis patient at baseline and at 3 months of biologic treatment. **(B)** Box graphical representation showing non-significant changes in CD3^+^CD4^+^ and CD3^+^CD4^-^ subsets in psoriasis patients after biologic treatment.

**Figure S4.** Relative distributions of Th1 and CD4^+^CCR6^+^ T subsets (Th17, Th17.1 and DN) were altered between controls (n = 10), responding patient’s baseline (n = 25) and after anti-IL17 biologic treatment (n = 25). **(A)** Relative distribution of CD4^+^ T cells (populations characterized as in Figure S1), as defined by CCR6, CXCR3 and CCR4 chemokine receptors expression. **(B)** Relative distribution of CD4^+^CCR6^+^ T cells (populations characterized as in Figure S1), as defined by CXCR3 and CCR4 chemokine receptors expression.

**Figure S5.** Analysis of CD3^+^CD4^+^ sub-population based on flow cytometry plots from peripheral blood mononuclear cells (PBMCs) from non-responding psoriasis patients was conducted (*n* = 5). Individual cell subsets were sub-gated based on the expression of CD3, CD4, CCR6, CCR4 and CXCR3 surface markers. **(A)** Representative flow cytometric plots showing non-significant changes in T cell phenotypes in a psoriasis patient at baseline and after anti-IL17 biologic therapy. **(B)** Box graphical representation showing non-significant changes in CD3^+^CD4^+^CCR6^+^ CCR4^+^ T (Th17), CD3^+^CD4^+^CCR6^+^CCR4^-^CXCR3^+^ (Th17.1), CD3^+^CD4^+^CCR6^-^CXCR3^+^ T (Th1), and CD3^+^CD4^+^CCR6^-^CXCR3^-^CCR4^+^ (Th2) cell subsets after anti-IL17 biologic therapy in peripheral blood from non-responding psoriasis patients. Percentages out of origin population from which each population is further sub-gated. Bar graphs showing the mean ± SD. ns non-significant by Wilcoxon signed rank test or paired t-test

**Figure S6.** CD4^+^CCR6^+^ T cell subpopulations express IL-17 in different percentages in psoriasis patients. **(A)** Representative flow cytometric plots showing a difference in IL-17-expression in CD4^+^CCR6^+^ T cell subpopulations, when further sub-gated by CCR4 expression in a psoriasis patient. **(B)** Representative flow cytometric plots showing differences in IL-17-expression in CD4^+^CCR6^+^CCR4^-^ T cell subpopulations, when further sub-gated by CXCR3 expression in a psoriasis patient. **(C)** Box graphical representation showing significant differences in IL-17 expression between Th17 and CCR6^+^CCR4^-^ cells and between Th17.1 and DN cells in psoriasis patients (n = 4). Bar graphs showing the mean ± SD. * p ≤ 0.05.
