# Supplementary material for: A sharp decrease of Th17, CXCR3+-Th17, and Th17.1 in peripheral blood is associated with an early anti-IL-17-mediated clinical remission in psoriasis
Source: Clin Exp Immunol. 2022 Aug 4;210(1):79–89. doi: 10.1093/cei/uxac069 (PMC9585551; doi:10.1093/cei/uxac069)
Supplement: uxac069_suppl_Supplementary_Table_S1 [file uxac069_suppl_supplementary_table_s1.docx]

Table S1. Laboratory values from patients (n = 30) at baseline and at 3 months.

|  | Baseline (10^9^/L) | 3 months (10^9^/L) |
| --- | --- | --- |
| Leukocyte Count | 7.73±1.63  4.82-11.8 | 7.31±1.52  4.80-10.80 |
| Lymphocytes | 2.08±0.59  1.04-3.89 | 1.99±0.43  1.00-3.21 |
| Monocytes | 0.57±0.12  0.33-0.79 | 0.51±0.12  0.28-0.83 |
| Eosinophiles | 0.22±0.12  0.00-0.47 | 0.23±0.13  0.00-0.52 |
| Basophiles | 0.03±0.02  0.00-0.09 | 0.03±0.03  0.00-0.10 |

Values are listed as mean ± SD and range.
