# Supplementary material for: A sharp decrease of Th17, CXCR3+-Th17, and Th17.1 in peripheral blood is associated with an early anti-IL-17-mediated clinical remission in psoriasis
Source: Clin Exp Immunol. 2022 Aug 4;210(1):79–89. doi: 10.1093/cei/uxac069 (PMC9585551; doi:10.1093/cei/uxac069)
Supplement: uxac069_suppl_Supplementary_Table_S2 [file uxac069_suppl_supplementary_table_s2.docx]

Table S2. Surface markers used to phenotypically characterize cell populations

| Phenotype | Cell population name |
| --- | --- |
| CD3^+^CD4^+^CCR6^+^CCR4^+^ | Th17 |
| CD3^+^CD4^+^CCR6^+^CCR4^+^CXCR3^+^ | CXCR3^+^-Th17 |
| CD3^+^CD4^+^CCR6^+^CCR4^+^CXCR3^-^ | CXCR3^-^-Th17 |
| CD3^+^CD4^+^CCR6^+^CCR4^-^CXCR3^+^ | Th17.1 |
| CD3^+^CD4^+^CCR6^+^CCR4^-^CXCR3^-^ | DN |
| CD3^+^CD4^+^CCR6^-^CXCR3^+^ | Th1 |
| CD3^+^CD4^+^CCR6^-^CXCR3^-^CCR4^+^ | Th2 |
| CD3^+^CD4^-^CCR6^+^CCR4^+^ | Tc17 |
| CD3^+^CD4^-^CCR6^+^CCR4^+^CXCR3^+^ | CXCR3^+^-Tc17 |
| CD3^+^CD4^-^CCR6^+^CCR4^-^CXCR3^+^ | Tc17.1 |
| CD3^+^CD4^-^CCR6^+^CCR4^-^CXCR3^-^ | DNc |
| CD3^+^CD4^-^CCR6^-^CXCR3^+^ | Tc1 |
| CD3^+^CD4^-^CCR6^-^CXCR3^-^CCR4^+^ | Tc2 |
