# Supplementary material for: A sharp decrease of Th17, CXCR3+-Th17, and Th17.1 in peripheral blood is associated with an early anti-IL-17-mediated clinical remission in psoriasis
Source: Clin Exp Immunol. 2022 Aug 4;210(1):79–89. doi: 10.1093/cei/uxac069 (PMC9585551; doi:10.1093/cei/uxac069)
Supplement: uxac069_suppl_Supplementary_Table_S3 [file uxac069_suppl_supplementary_table_s3.docx]

Table S3. Simple linear regressions assessing the effect of prior treatment on inhibition of cell subsets.

| Simple Linear Regression Number | Dependent Variable | Independent Variable (Prior therapy group/Reference group) | β coefficient | 95% CI | p value |
| --- | --- | --- | --- | --- | --- |
| 1 | Th17_Dif | csDMARDs/naïve | 0.8542 | -1.694, 3.402 | 0.4924 |
|  |  | apremilast/naïve | 0.08 | -3.256, 3.416 | 0.9606 |
|  |  | anti-TNF/naïve | -0.1383 | -3.183, 2.907 | 0.9254 |
|  |  | anti-IL12/23/naive | 0.2517 | -2.793, 3.297 | 0.8649 |
| 2 | CXCR3^+^-Th17_Dif | csDMARDs/naïve | 15.86 | -4.446, 36.17 | 0.1189 |
|  |  | apremilast/naïve | 26.80 | 0.2103, 53.39 | 0.0484 |
|  |  | anti-TNF/naïve | 7.750 | -16.52, 32.02 | 0.5130 |
|  |  | anti-IL12/23/naive | -2.900 | -27.17, 21.37 | 0.8057 |
| 3 | Th17.1_Dif | csDMARDs/naïve | -3.829 | -21.38, 13.72 | 0.6539 |
|  |  | apremilast/naïve | 16.60 | -6.374, 39.57 | 0.1474 |
|  |  | anti-TNF/naïve | -1.642 | -22.61, 19.33 | 0.8719 |
|  |  | anti-IL12/23/naive | 1.233 | -19.74, 22.21 | 0.9036 |
